# Submucosal uterine leiomyoma with complete cystic degeneration mimicking an empty gestational sac: a case report and literature review

**DOI:** 10.3389/fmed.2026.1858581

**Published:** 2026-06-26

**Authors:** Shunlin Lin, Di An, Zihua Xu

**Affiliations:** Department of Gynecology, Huidong County People’s Hospital, Huizhou, Guangdong, China

**Keywords:** anembryonic pregnancy, case report, cystic degeneration, hysteroscopy, immunohistochemistry, leiomyoma, submucosal myoma

## Abstract

**Background:**

Uterine leiomyomas represent the most frequent benign pelvic tumors in women. Although various degenerative changes can occur, the complete cystic transformation of a submucosal leiomyoma—resulting in a large, fluid-filled intracavitary mass—is an exceptional clinical entity. Such presentations often pose diagnostic dilemmas by mimicking other conditions, notably an empty gestational sac.

**Case presentation:**

A 42-year-old parous woman (G3P2) presented with irregular vaginal bleeding lasting for 2 months, accompanied by recent-onset lower abdominal pain. Initial transvaginal ultrasonography demonstrated a large (65 mm × 45 mm) unilocular cystic lesion within the uterine cavity, raising the suspicion of an anembryonic pregnancy. Serial serum β-hCG testing yielded negative results. Subsequent hysteroscopy revealed a giant, smooth-walled cystic tumor originating from the posterior wall, which was entirely excised via electrosurgical resection. Preliminary histological evaluation indicated a mesenchymal neoplasm with focal proliferative features. A comprehensive immunohistochemical panel—showing positivity for SMA, Desmin, h-Caldesmon, CD10, and FH; negative 2SC; wild-type p53; a Ki-67 index near 5%; and scarce PHH3-positive cells—ultimately established the diagnosis of a completely degenerated cystic submucosal leiomyoma with focal proliferative activity. Follow-up imaging at 3 months confirmed the absence of recurrence.

**Conclusion:**

This case underscores that a completely cystic submucosal leiomyoma warrants consideration in the differential diagnosis of intracavitary cystic lesions, even when imaging findings are suggestive of an empty gestational sac. A negative serum β-hCG is a pivotal diagnostic clue. Hysteroscopy is invaluable for both direct visualization and therapeutic resection, while immunohistochemistry is essential for definitive diagnosis—particularly when initial histopathology is inconclusive.

## 1 Introduction

Uterine leiomyomas arise from the smooth muscle cells of the myometrium and represent the most common benign tumor of the female reproductive system. Based on their anatomical relationship to the uterine wall, they are broadly categorized as submucosal, intramural, or subserosal, with a notably high prevalence among women of reproductive age (1). While the vast majority present as solid masses, a minority undergo degenerative changes. Cystic degeneration is a comparatively uncommon morphological variant, thought to arise from intratumoral ischemia leading to necrosis and progressive liquefaction (2). When a submucosal leiomyoma undergoes extensive cystic transformation and protrudes entirely into the uterine cavity, it may appear on imaging as a solitary intracavitary cystic structure, creating a formidable diagnostic challenge. The differential diagnosis for such a finding is broad, encompassing entities including an empty gestational sac, cystic endometrial polyps, cystic endometrial carcinoma, uterine sarcoma, and other rare benign mesenchymal tumors (3).

The rarity of this case stems from its unusual presentation as a large, purely cystic intracavitary lesion, which can easily mislead clinicians toward pregnancy-related complications (2, 4, 5). The present report delineates a comprehensive diagnostic pathway, transitioning from an initial sonographic misinterpretation to a definitive diagnosis via hysteroscopic intervention and rigorous pathological assessment. Hysteroscopy proved indispensable by facilitating both direct morphological evaluation and simultaneous therapeutic resection (6). Additionally, the identification of “focal proliferative activity” highlights the necessity of distinguishing this entity from cellular leiomyomas and other variants—a crucial step for determining biological behavior and guiding appropriate follow-up (7).

With these considerations in mind, this report aims to sharpen clinicians’ awareness of atypical leiomyoma presentations, broaden the differential diagnostic framework for intracavitary cystic lesions, and reaffirm the irreplaceable role of histopathological examination as the gold standard in gynecologic diagnosis.

## 2 Case description

### 2.1 Patient information

A 42-year-old woman (gravida 3, para 2) attended our outpatient clinic with a chief complaint of irregular vaginal bleeding persisting for approximately 2 months and lower abdominal pain of three days’ duration. The bleeding was intermittent, dark red in color, and lighter in volume than her usual menstrual flow; she denied dysmenorrhea or the passage of clots. Prior menstrual cycles had been regular. Three days before presentation, she developed a dull aching sensation in the lower abdomen without any identifiable precipitating factor. Her past medical, surgical, and family histories were unremarkable.

### 2.2 Clinical findings

**FIGURE 1:**
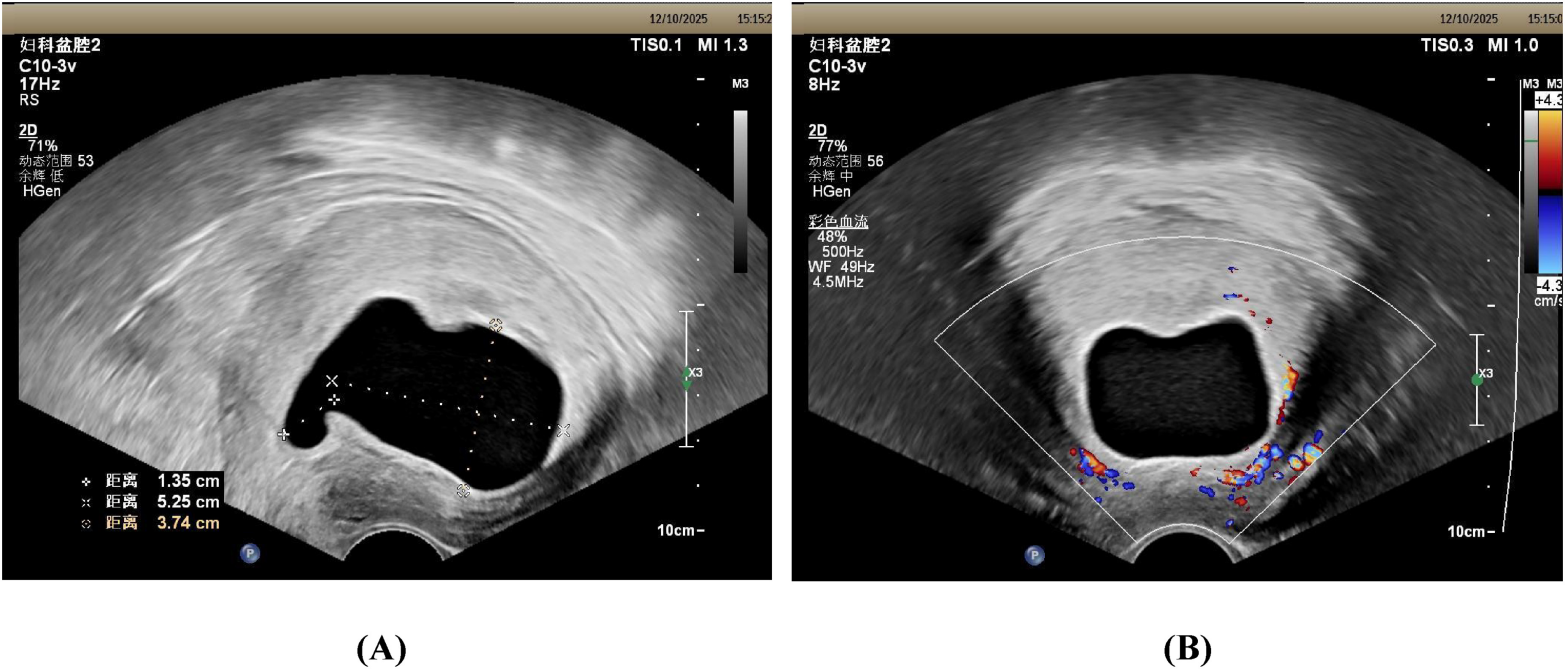
Preoperative transvaginal ultrasonographic findings. **(A)** B-mode ultrasound demonstrating a large, unilocular, anechoic cystic lesion (approximately 65 mm × 45 mm) with a thickened wall, occupying the uterine cavity and initially suspected to represent an empty gestational sac. **(B)** Color Doppler imaging revealing minimal peripheral vascularity surrounding the lesion, with no significant internal blood flow detected.

Transvaginal color Doppler ultrasonography performed in the outpatient setting disclosed a large cystic lesion measuring approximately 65 mm by 45 mm within the uterine cavity (Figure 1A). The lesion had a relatively thick wall and was filled with clear fluid. Color Doppler interrogation demonstrated only minimal peripheral vascularity, with no appreciable internal blood flow (Figure 1B). The initial sonographic impression raised the possibility of an empty gestational sac, though other etiologies could not be excluded. A serum β-hCG level obtained at the same visit returned below 0.1 IU/L. On the basis of these findings, the patient was admitted for further evaluation.

### 2.3 Timeline

Key clinical events are summarized in Table 1.

**TABLE 1:** Timeline of key clinical events.

Timeline	Event
2 months prior to admission	Onset of intermittent, irregular vaginal bleeding.
3 days prior to admission	Emergence of lower abdominal pain.
Day of admission	Outpatient transvaginal ultrasound identified a large intracavitary cystic lesion (65 mm × 45 mm); serum β-hCG < 0.1 IU/L. Patient admitted for further evaluation.
Hospital day 1	Completion of inpatient workup including blood tests, coagulation profile, and tumor markers (CA125, CA199, CEA, AFP, HE4)—all within normal limits. Repeat β-hCG < 0.1 IU/L.
Hospital day 2	Hysteroscopic resection performed; a large cystic mass was visualized and excised. Intraoperative irrigation volume: 12,000 mL; furosemide administered prophylactically.
Hospital day 3	Uneventful recovery; patient discharged.
Postoperative day 7	Histopathological examination confirmed cystic leiomyoma with focal proliferative features; immunohistochemistry performed for definitive characterization.
3 months post-surgery	Follow-up ultrasonography showed no residual intracavitary mass.

### 2.4 Diagnostic evaluation, therapeutic approach, and clinical outcome

Upon admission, the patient underwent a thorough diagnostic evaluation. Routine laboratory investigations—including complete blood count, coagulation profile, liver and renal function tests, and vaginal discharge analysis—were all within normal limits. Serum tumor markers (CA125, CA199, CEA, AFP, and HE4) were similarly unremarkable. A repeat serum β-hCG confirmed a level below 0.1 IU/L. Given the uncertain nature of the intracavitary lesion, hysteroscopy with diagnostic curettage was scheduled.

Intraoperatively, a large, spherical mass enveloped by endometrial tissue was identified within the uterine cavity. Following diagnostic curettage, repeat hysteroscopic inspection revealed that the endometrial covering over the lesion could be stripped away. Puncture of the mass confirmed its cystic nature, yielding clear, pale yellow fluid. After aspiration of the cystic contents, the mass decreased in volume but continued to occupy a substantial portion of the cavity. The lesion was subsequently resected using a hysteroscopic electrosurgical loop. The base of the mass had a relatively clear demarcation from the myometrium, with approximately one-third extending into the muscular layer. The procedure lasted approximately 1 h, with a total irrigation volume of 12,000 mL of isotonic saline and an outflow of approximately 9,000 mL. To mitigate the risk of fluid overload, furosemide was administered intravenously (20 mg at 6,000 mL and an additional 10 mg at 10,000 mL of irrigation). Intraoperative blood loss was minimal. The patient remained hemodynamically stable throughout and was ambulatory by the first postoperative day.

Initial histopathological evaluation suggested a mesenchymal tumor exhibiting focal proliferative activity, with malignancy unable to be definitively excluded at that stage. Following further pathological consultation and a comprehensive immunohistochemical panel, the diagnosis was refined to a uterine leiomyoma exhibiting cystic degeneration with focal proliferative features. The immunohistochemical staining results were as follows: Smooth Muscle Actin (SMA): positive (+); Desmin: positive (+); h-Caldesmon: positive (+); CD10: positive (+); Fumarate Hydratase (FH): positive (+); 2-Succinylcysteine (2SC): negative (-); p53: focally positive, wild-type staining pattern; Ki-67 proliferative index: approximately 5%; Phosphohistone H3 (PHH3): rare positive cells.

**FIGURE 2:**
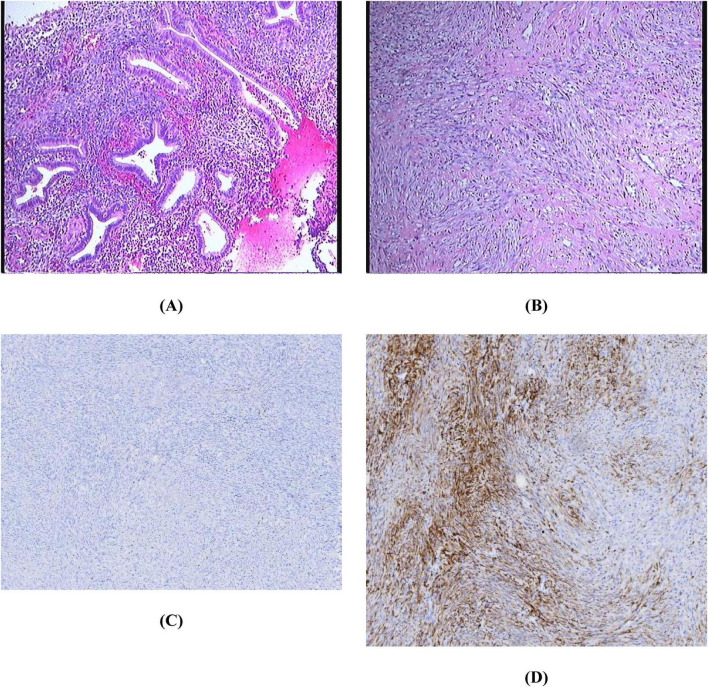
Histopathological and immunohistochemical findings. **(A)** Low-power photomicrograph (H&E stain, ×40) demonstrating the tumor wall composed of spindle-shaped cells with areas of cystic degeneration. **(B)** High-power photomicrograph (H&E stain, ×200) revealing bland spindle cells in interlacing fascicles with focal areas of increased cellularity, but without significant nuclear atypia or atypical mitotic figures. **(C)** Immunohistochemical staining for PHH3 showing rare positive cells, indicating a low mitotic index consistent with benign behavior. **(D)** Immunohistochemical staining for CD10 demonstrating focal positive expression, which can be observed in uterine leiomyomas.

These findings were consistent with a smooth muscle origin. Positivity for SMA, Desmin, and h-Caldesmon confirmed the smooth muscle phenotype. Retained FH expression and negative 2SC staining effectively excluded HLRCC-associated leiomyoma. The wild-type p53 pattern, low Ki-67 index (∼5%), and rare PHH3-positive cells collectively supported a benign diagnosis and were not suggestive of leiomyosarcoma or other high-grade malignancies. Notably, focal CD10 positivity was observed; while CD10 is traditionally associated with endometrial stromal tumors, its focal expression in uterine leiomyomas is well-documented and does not alter the diagnosis when the overall immunophenotype is consistent with smooth muscle differentiation (Figure 2).

**FIGURE 3:**
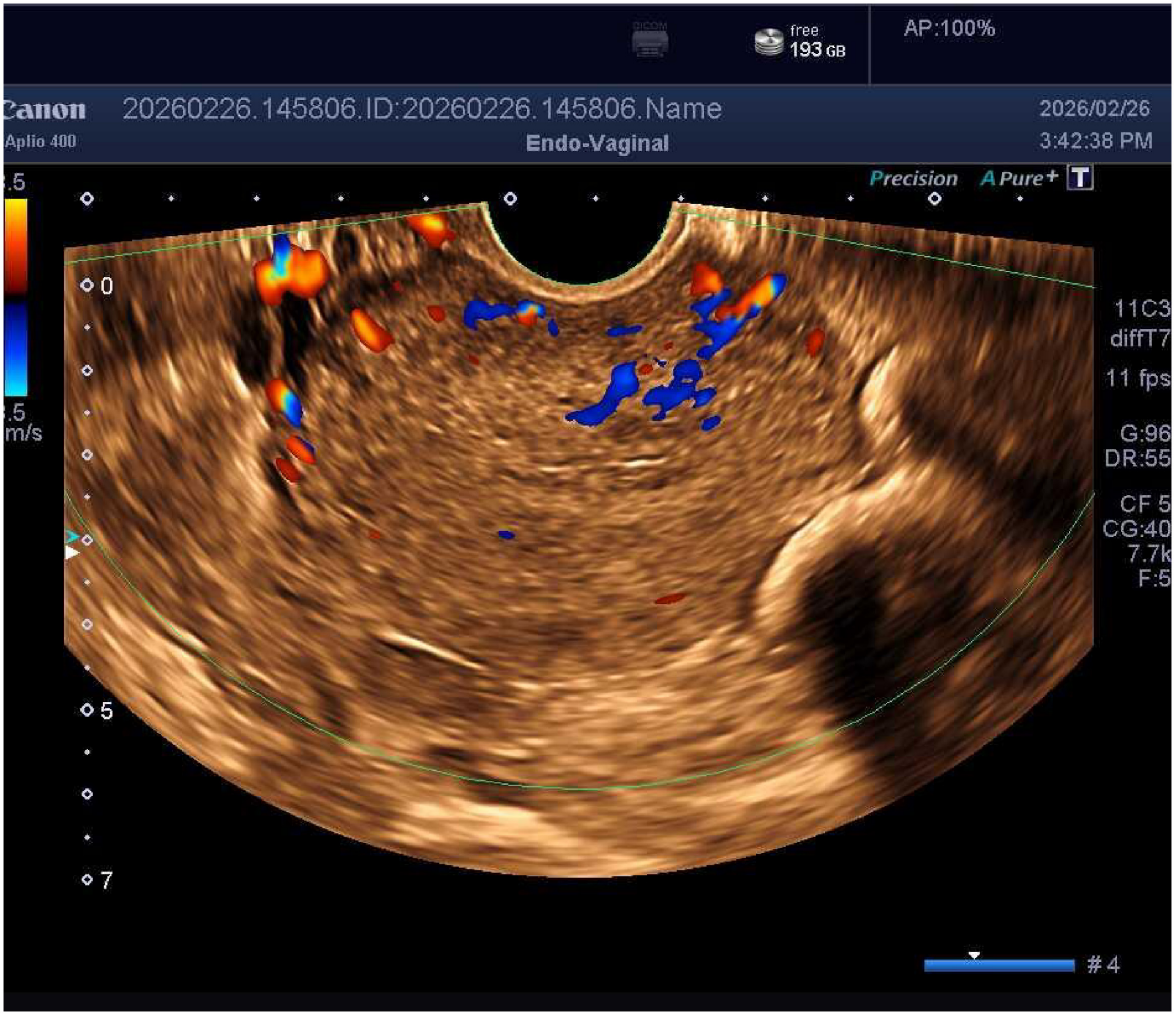
Postoperative follow-up transvaginal color Doppler ultrasound at 3 months demonstrating no residual intracavitary mass, with a normal endometrial echo pattern.

At the 3-month follow-up, transvaginal ultrasonography demonstrated an endometrial thickness of 9 mm with heterogeneous echotexture but no evidence of residual intracavitary mass. Both adnexal regions appeared normal (Figure 3).

## 3 Discussion

From an imaging perspective, a unilocular cystic leiomyoma can closely mimic an anembryonic pregnancy. Critical sonographic differentiators include the absence of embryonic structures and the presence of a thickened, avascular wall—though these features alone are insufficient for a definitive diagnosis. Cystic endometrial pathologies, such as cystic polyps or hyperplasia, typically manifest as multilocular or honeycomb-like structures rather than a single dominant cyst, providing a useful distinguishing feature.

Cystic degeneration in uterine leiomyomas is a recognized phenomenon; however, its manifestation as a large, unilocular, clear-fluid-filled intracavitary mass remains exceedingly uncommon and can be readily mistaken for pregnancy-related entities such as an empty gestational sac (2). Most documented cases of cystic leiomyomas involve multilocular cystic structures with a complex imaging appearance, often closely associated with the myometrium (8). The present case, in contrast, exhibited a unilocular cystic lesion with clear fluid located entirely within the uterine cavity—a pattern that diverges markedly from the typical imaging characteristics of cystic leiomyomas and contributed substantially to the initial misdiagnosis. While submucosal leiomyomas are common, their presentation as a giant cystic mass rather than a solid growth pattern is distinctly unusual, compounding the preoperative diagnostic difficulty (9).

The histopathological finding of “focal proliferative activity” raised the critical need to differentiate this lesion from cellular leiomyoma and smooth muscle tumors of uncertain malignant potential (STUMP) (9). Cellular leiomyomas occupy a diagnostic gray zone, with biological behavior that is not fully predictable, necessitating rigorous pathological assessment based on criteria such as mitotic count, cellular atypia, and the presence or absence of coagulative tumor cell necrosis (10, 11). To ensure diagnostic precision and rule out rare malignant or syndromic differentials, an extensive immunohistochemical workup was performed in accordance with institutional protocols for atypical mesenchymal tumors. The observed focal proliferative activity denotes localized regions of increased cellularity lacking significant nuclear atypia or abnormal mitotic figures, aligning with a benign yet biologically active leiomyoma variant. In our case, the immunohistochemical profile—positivity for smooth muscle markers (SMA, Desmin, h-Caldesmon), focal CD10 expression, retained FH expression with negative 2SC staining, a wild-type p53 staining pattern, a low Ki-67 proliferative index (∼5%), and rare PHH3-positive cells—collectively confirmed the benign smooth muscle origin of the tumor. This underscores the indispensable role of thorough histopathological evaluation, supplemented by immunohistochemistry, in discriminating benign from malignant uterine smooth muscle tumors (12) (Figure 2).

Unlike cases of cystic adenomyosis or adnexal cystic neoplasms, the uterine myometrial origin of the lesion in our patient was conclusively established through direct hysteroscopic visualization and subsequent pathological confirmation, thereby avoiding misdiagnosis as an ovarian tumor or other pelvic cystic pathology (13, 14). The role of hysteroscopy in this case extended beyond mere diagnosis; it served as the definitive therapeutic modality, enabling complete resection of the lesion in a single procedure. This approach not only spared the patient from more invasive surgery but also provided direct tissue sampling for histopathological analysis—a dual advantage that is particularly valuable in the management of unusual intracavitary lesions (15–17).

Notably, focal CD10 positivity was observed in the immunohistochemical panel. CD10 is traditionally considered a marker for endometrial stromal tumors, and its expression in a smooth muscle tumor may initially raise diagnostic concern. However, focal CD10 expression has been well-documented in uterine leiomyomas, particularly in cellular variants and those with degenerative changes (18–20). In the context of strong positivity for smooth muscle markers (SMA, Desmin, h-Caldesmon) and the overall morphological features, CD10 positivity alone does not alter the diagnosis. Additionally, the retained FH expression and negative 2SC staining are important findings that exclude HLRCC-associated leiomyoma—a rare hereditary condition characterized by FH deficiency and an elevated risk of renal cell carcinoma. Screening for this syndrome is clinically relevant, particularly in younger patients with multiple leiomyomas (18).

We acknowledge that pelvic MRI could have provided additional characterization of this atypical cystic lesion, particularly to exclude rare malignant or subserosal pathologies (3, 4, 9). Nevertheless, our decision to proceed with hysteroscopic exploration was based on an integrated assessment of clinical symptoms, laboratory data, and preoperative ultrasound findings. First, transvaginal ultrasonography revealed a thick-walled, unilocular, anechoic cyst with no internal vascularity — features that, while misleading, showed no signs of solid or invasive malignancy. Second, a full panel of serum tumor markers (CA125, CA199, CEA, AFP, HE4) was entirely normal, further lowering the pretest probability of an aggressive uterine malignancy. Third, the patient’s clinical picture —2 months of persistent irregular bleeding with repeatedly negative β-hCG — was most consistent with either a resolved anembryonic pregnancy or a benign intracavitary lesion, both of which require histologic sampling for definitive diagnosis. Hysteroscopy offered direct visualization and single-session resection, avoiding diagnostic delays, extra costs, and unnecessary investigations associated with MRI; we therefore selected it as the primary intervention (6, 16, 19). The patient received detailed preoperative counseling about the theoretical risk of retrograde tumor spread via distention fluid and provided formal informed consent. At our institution, urgent same-day MRI is not available for routine outpatient workups, and the patient’s active bleeding and anxiety supported a streamlined diagnostic approach. We agree that MRI remains strongly indicated when deep myometrial invasion or an overtly malignant imaging phenotype is suspected.

The present case also illustrates the inherent limitations of ultrasound as a sole diagnostic modality for unusual intracavitary lesions. The sonographic features—a large, unilocular, anechoic cystic structure with a thickened wall and absent internal vascularity—are features shared by both an empty gestational sac and a cystic leiomyoma. In the absence of a positive β-hCG, the clinical presentation in this patient was particularly deceptive. The fact that the patient’s symptoms had persisted for approximately 2 months without resolution, combined with the negative β-hCG, should have prompted earlier consideration of a non-gestational etiology. This case serves as a practical reminder that whenever an intracavitary cystic structure is encountered, pregnancy must first be excluded with serum β-hCG; thereafter, a broad differential diagnosis should be maintained, and tissue sampling should be pursued when the diagnosis remains uncertain (21).

We acknowledge certain limitations in this report. The follow-up duration of 3 months is relatively brief; extended surveillance is therefore planned to monitor for potential recurrence, despite the reassuring benign pathology and complete macroscopic resection. Additionally, the inability to capture intraoperative hysteroscopic images due to equipment constraints represents a recognized shortcoming, which we have sought to mitigate through comprehensive histopathological documentation.

## 4 Conclusion

This case draws attention to a rare but clinically important presentation of submucosal uterine leiomyoma with complete cystic degeneration, which closely mimicked an empty gestational sac on transvaginal ultrasonography. The diagnostic process was guided by serial negative β-hCG measurements and ultimately resolved through hysteroscopic exploration and comprehensive histopathological and immunohistochemical analysis. The immunohistochemical profile—particularly the combination of smooth muscle marker positivity, retained FH expression, negative 2SC, wild-type p53, low Ki-67, and rare PHH3-positive cells—was instrumental in establishing a definitive benign diagnosis and excluding malignancy. This report reinforces the value of maintaining a broad differential diagnosis for intracavitary cystic lesions, the indispensable role of hysteroscopy in their management, and the necessity of thorough pathological evaluation when initial findings are equivocal.

## Data Availability

The original contributions presented in this study are included in this article/supplementary material, further inquiries can be directed to the corresponding author.

## References

[1] Pritts EA. Uterine leiomyomas and reproduction. *Obstet Gynecol.* (2025) 145:39–45. 10.1097/AOG.0000000000005748 39326048

[2] Nishio E Sakabe Y Fujii T. Rare recurrence of a multilocular cystic leiomyoma following myomectomy. *Fujita Med J.* (2023) 9:160–2. 10.20407/fmj.2022-013 37234393 PMC10206892

[3] Yacoub JH Clark JA Paal EE Manning MA. Approach to cystic lesions in the abdomen and pelvis, with radiologic-pathologic correlation. *Radiographics.* (2021) 41:1368–86. 10.1148/rg.2021200207 34469214 PMC8415047

[4] Tihy M Bucau M. Cystic tumors: diagnosis and pathological challenges. *Ann Pathol.* (2025) 45:480–8. 10.1016/j.annpat.2025.07.004 40912986

[5] Koch E Torsten U Mecke H Richter R Hellmeyer L Nohe G et al. Patients’ subjective assessment as a decisive predictor of malignancy in pelvic masses. *J Psychosom Obstet Gynaecol.* (2022) 43:273–8. 10.1080/0167482X.2020.1850684 33252280

[6] Castaldi MA Castaldi SG. Office hysteroscopic treatment of vaginal bleeding and related pain after supracervical hysterectomy: a case report. *SAGE Open Med Case Rep.* (2025) 13:2050313X251407045. 10.1177/2050313X251407045 41458575 PMC12743159

[7] Wuori D Chapel DB. Uterine leiomyoma with bizarre nuclei. *Int J Gynecol Cancer.* (2024) 34:958–9. 10.1136/ijgc-2023-004560 37923318

[8] Ashindoitiang JA Canice Nwagbara VI Edet EE Ugbem TI Ukam JS Asuquo ME. Large subserous uterine leiomyoma presenting as intraabdominal tumor: a case report. *Rare Tumors.* (2024) 16:20363613241285089. 10.1177/20363613241285089 39290295 PMC11406654

[9] Lin Y Wu RC Huang YL Chen K Tseng SC Wang CJ et al. Uterine fibroid-like tumors: spectrum of MR imaging findings and their differential diagnosis. *Abdom Radiol.* (2022) 47:2197–208. 10.1007/s00261-022-03431-6 35347386

[10] Qu Q Ma Z Liu G Zhang X Xu C Wu X. Uterine sarcomas: computed tomography and magnetic resonance imaging findings. *J Cancer Res Ther.* (2025) 21:1207–12. 10.4103/jcrt.jcrt_473_25 41474243

[11] Brunet A Verkarre V Le Frère Belda MA. A special uterine leiomyoma. *Ann Pathol.* (2020) 40:180–4. 10.1016/j.annpat.2020.02.021 32192807

[12] Ye Z Jiang Y Yan K Yu C. Uterine torsion with degeneration and infarction of giant leiomyoma in a postmenopausal woman: a case report. *Medicine.* (2023) 102:e35964. 10.1097/MD.0000000000035964 37960802 PMC10637470

[13] Li C Xu Y Cong L. Laparoscopic treatment of a large cystic adenomyosis of the uterus: a case report. *Int J Surg Case Rep.* (2020) 71:179–82. 10.1016/j.ijscr.2020.04.084 32460088 PMC7251317

[14] Hashizume K Toyoshima M Shiraishi T Ueno Y Yamamoto A Kawase R et al. Carcinosarcoma of the uterus, derived from subserous cystic adenomyosis, presenting as an acute abdomen: a case report and review of the literature. *Gynecol Oncol Rep.* (2023) 45:101139. 10.1016/j.gore.2023.101139 36747897 PMC9898588

[15] Quilon M Glucksman A Emmanuel G Greenstein J Hahn B. Female with vaginal bleeding. *Clin Pract Cases Emerg Med.* (2020) 4:636–7. 10.5811/cpcem.2020.8.48627 33217295 PMC7676798

[16] Jeong N Cho A Koo YJ Ahn JW Park H Lee ES et al. Clinical practice in office hysteroscopy. *Obstet Gynecol Sci.* (2025) 68:175–85. 10.5468/ogs.24234 40181699 PMC12104621

[17] Khoiwal K Zaman R Bahurupi Y Gaurav A Chaturvedi J. Comparison of vaginoscopic hysteroscopy and traditional hysteroscopy: a systematic review and meta-analysis. *Int J Gynaecol Obstet.* (2024) 164:47–55. 10.1002/ijgo.14902 37306153

[18] Shivarajpur A Kohen B. Vaginal bleeding in a peri-menopausal woman. *Pocus J.* (2024) 9:9–10. 10.24908/pocus.v9i1.16621 38681151 PMC11044943

[19] Breen B. A guide to hysteroscopy. *Br J Nurs.* (2022) 31:1–6. 10.12968/bjon.2022.31.Sup18.S1 39189942

[20] Shrestha E Mishra A. Zosteriform pilar leiomyoma associated with uterine leiomyoma: a case report. *Clin Case Rep.* (2023) 11:e6904. 10.1002/ccr3.6904 36777791 PMC9900235

[21] Okohue JE. Overview of hysteroscopy. *West Afr J Med.* (2020) 37:178–82. 32150637

